# Unsupervised machine learning based on clinical factors for the detection of coronary artery atherosclerosis in type 2 diabetes mellitus

**DOI:** 10.1186/s12933-022-01700-8

**Published:** 2022-11-28

**Authors:** Yu Jiang, Zhi-Gang Yang, Jin Wang, Rui Shi, Pei-Lun Han, Wen-Lei Qian, Wei-Feng Yan, Yuan Li

**Affiliations:** 1 Department of Radiology, West China Hospital, Sichuan University, 37# Guo Xue Xiang, Chengdu, 610041 Sichuan China; 2 West China Biomedical Big Data Centre, West China Hospital, Sichuan University, Chengdu, China

**Keywords:** Machine learning, Coronary atherosclerosis, Diabetes mellitus, Coronary computed tomography angiography

## Abstract

**Background:**

Coronary atherosclerosis can lead to serious cardiovascular events. In type 2 diabetes (T2DM) patients, the effects of clinical factors on coronary atherosclerosis have not been fully elucidated. We used a clustering method to distinguish the population heterogeneity of T2DM and the differences in coronary atherosclerosis evaluated on coronary computed tomography angiography (CCTA) among groups and to facilitate clinical management.

**Methods:**

Clinical data from 1157 T2DM patients with coronary atherosclerosis who underwent CCTA in our hospital from January 2018 to September 2021 were retrospectively collected. The coronary artery segment plaque type and stenosis, the number of involved vessels, the segment involvement score (SIS) and the segment stenosis score (SSS) were evaluated and calculated. Unsupervised clustering analysis based on clinical information was used (cluster 1: n = 463; cluster 2: n = 341; cluster 3: n = 353). The association of coronary plaque characteristics with cluster groups was evaluated.

**Results:**

The clinical data among the three groups were different in several aspects: (1) Cluster 1 had the least male patients (41.7%), the lowest proportion of patients with smoking (0%) or alcohol history (0.9%), and the lowest level of serum creatinine (74.46 ± 22.18 µmol/L); (2) Cluster 2 had the shortest duration of diabetes (7.90 ± 8.20 years) and was less likely to be treated with diabetes (42.2%) or statins (17.6%) and (3) Cluster 3 was the youngest (65.89 ± 10.15 years old) and had the highest proportion of male patients (96.6%), the highest proportion of patients with smoking (91.2%) and alcohol (59.8%) history, the highest level of eGFR (83.81 ± 19.06 ml/min/1.73m^2^), and the lowest level of HDL-C (1.07 ± 0.28 mmol/L).

The CCTA characteristics varied with different clusters: (1) Cluster 1 had the largest number of segments with calcified plaques (2.43 ± 2.46) and the least number of segments with mixed plaques (2.24 ± 2.59) and obstructive stenosis (0.98 ± 2.00); (2) Cluster 1 had the lowest proportion of patients with mixed plaques (68%) and obstructive stenosis (32.2%); (3) Cluster 3 had more segments with noncalcified plaques than cluster 1 (0.63 ± 1.02 vs 0.40 ± 0.78, P < 0.05) and the highest proportion of patients with noncalcified plaques (39.9%) and (4) There was no significant difference in the extent of coronary plaques among the three clusters.

**Conclusions:**

The unsupervised clustering method could address T2DM patients with heterogeneous clinical indicators and identify groups with different types of coronary plaque and degrees of coronary stenosis. This method has the potential for patient stratification, which is essential for the clinical management of T2DM patients with coronary atherosclerosis.

## Background

Diabetes is a serious threat to public health. According to data from the International Diabetes Federation (IDF), the number of people with diabetes has reached 536 million worldwide, and it is estimated that this number will rise to 783 million by 2045 [1]. Type 2 diabetes mellitus (T2DM) is the most common type of diabetes. Cardiovascular disease is a common complication of T2DM and is the leading cause of morbidity and mortality among diabetes patients [2]. The presence of T2DM is associated with an increased risk of cardiovascular disease and cardiovascular events [3–6].

Coronary atherosclerosis is a major cardiovascular disease. The assessment of blood pressure, plasma lipid levels and other risk factors could be used to guide the management of atherosclerotic cardiovascular disease [2]. T2DM has traditionally been considered a risk factor for atherosclerosis and can accelerate the progression of coronary atherosclerosis [7, 8]. T2DM is often accompanied by other risk factors [1, 9]. Although the risk factors for cardiovascular disease have been elucidated separately [10–12], the different clinical features of T2DM patients have varying influences on coronary atherosclerosis [13–15]. The comprehensive effect of the clinical features of T2DM on coronary atherosclerosis still needs further study.

Machine learning methods have been widely used in cardiovascular disease research [16, 17]. An unsupervised machine learning approach has been applied to clarify the heterogeneity of coronary artery disease, indicating the feasibility of this method to identify important subpopulations based on clinical data [17]. However, few studies have focused on the exploration of unsupervised machine learning in distinguishing the clinical heterogeneity of T2DM and the relationships between the subgroups and coronary atherosclerosis characteristics. Accordingly, we used unsupervised machine learning to analyze the heterogeneity of T2DM patients based on clinical indicators and to clarify the comprehensive effect of clinical factors on coronary atherosclerosis characteristics detected on coronary computed tomography angiography (CCTA), which may facilitate individualized clinical management.

## Methods

This retrospective study was approved by the Biomedical Research Ethics Committee of our hospital, and written informed consent was waived.

### Study cohort

Between January 2018 and November 2021, T2DM patients with coronary plaque detected on CCTA in our hospital were retrospectively reviewed. The exclusion criteria were as follows: patients with a history of coronary artery bypass grafting or stenting before CCTA scanning; CCTA image quality that was too poor for coronary artery plaque assessment; incomplete clinical information; and severe renal failure [estimated glomerular filtration rate (eGFR) < 30 mL/min/1.73 m^2^]. Finally, 1157 patients with T2DM were included in the study.

### CT scanning protocols

The CCTA examinations were performed using a GE CT scanner (Revolution CT, GE Healthcare, Waukesha, WI, USA) or Siemens CT scanner (SOMATOM Definition FLASH, Siemens Medical Solutions, Forchheim, Germany; or SOMATOM Definition, Siemens Medical Solutions, Forchheim, Germany). A bolus of nonionized contrast agent was intravenously injected. The CCTA scanning ranged from the tracheal bifurcation to 20 mm below the inferior cardiac apex. The parameters were as follows: For Siemens CT scanners, tube voltage of 100–120 kV, tube current of 220 mAs; collimation, 64/128 × 0.5 mm. For the GE Revolution CT, the tube voltage was set to 120 kV, the tube current was automatically adjusted, and the image slice thickness was reconstructed to 0.625 mm. A prospective or retrospective electrocardiogram-gated protocol was used for CCTA image acquisition.

### CCTA analysis

**Fig. 1:**
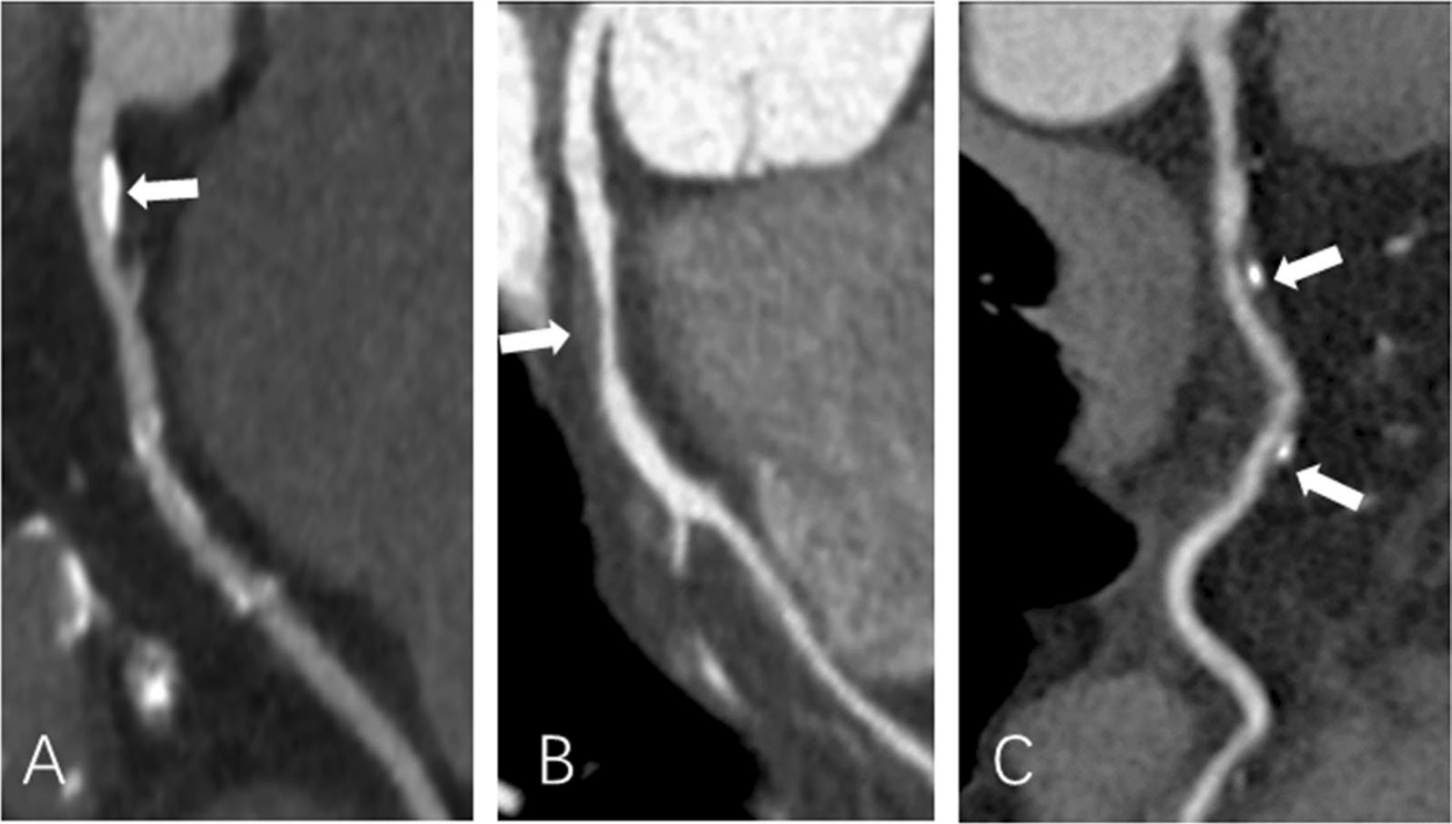
Representative coronary computed tomography angiography images of **A** calcified plaque, **B** noncalcified plaque, and **C** mixed plaque

The CCTA analysis included assessment of plaque type and stenosis of the coronary artery segment and calculation of the segment involvement score (SIS) and segment stenosis score (SSS). By visual evaluation, coronary plaques were classified into three types: calcified plaque, noncalcified plaque and mixed plaque (Fig. 1). Calcified plaque was defined as plaque containing only calcified components; noncalcified plaque was defined as plaque without any calcification that manifested as plaque with computed tomographic density lower than the contrast-enhanced coronary lumen; calcification with noncalcified components shown in a single plaque was defined as mixed plaque [18]. The stenosis degree was estimated according to the Coronary Artery Disease-Reporting and Data System (CAD-RADS) [19]: score 0 (absence of plaque), score 1 (luminal stenosis < 25%), score 2 (25–49% luminal stenosis), score 3 (50–69% luminal stenosis), score 4 (70–99% luminal stenosis), or score 5 (total occlusion). Obstructive stenosis was defined as any presence of stenosis > 50%, and obstructive disease was defined as the presence of obstructive stenosis. The SIS was defined as the number of coronary artery segments observed with plaques. The SSS was defined as the sum of the stenosis scores of the relevant stenosis grades of all segments. According to the modified American Heart Association standard [20], the assessment of the coronary artery included four main vessels (left main artery, left anterior descending coronary artery, left coronary circumflex artery and right coronary artery) and 16 segments. Two cardiovascular radiologists who were blinded to the clinical information of the patients evaluated the images independently. The two observers reached a consensus by discussion when there were divergences.

### Unsupervised machine learning

**Fig. 2:**
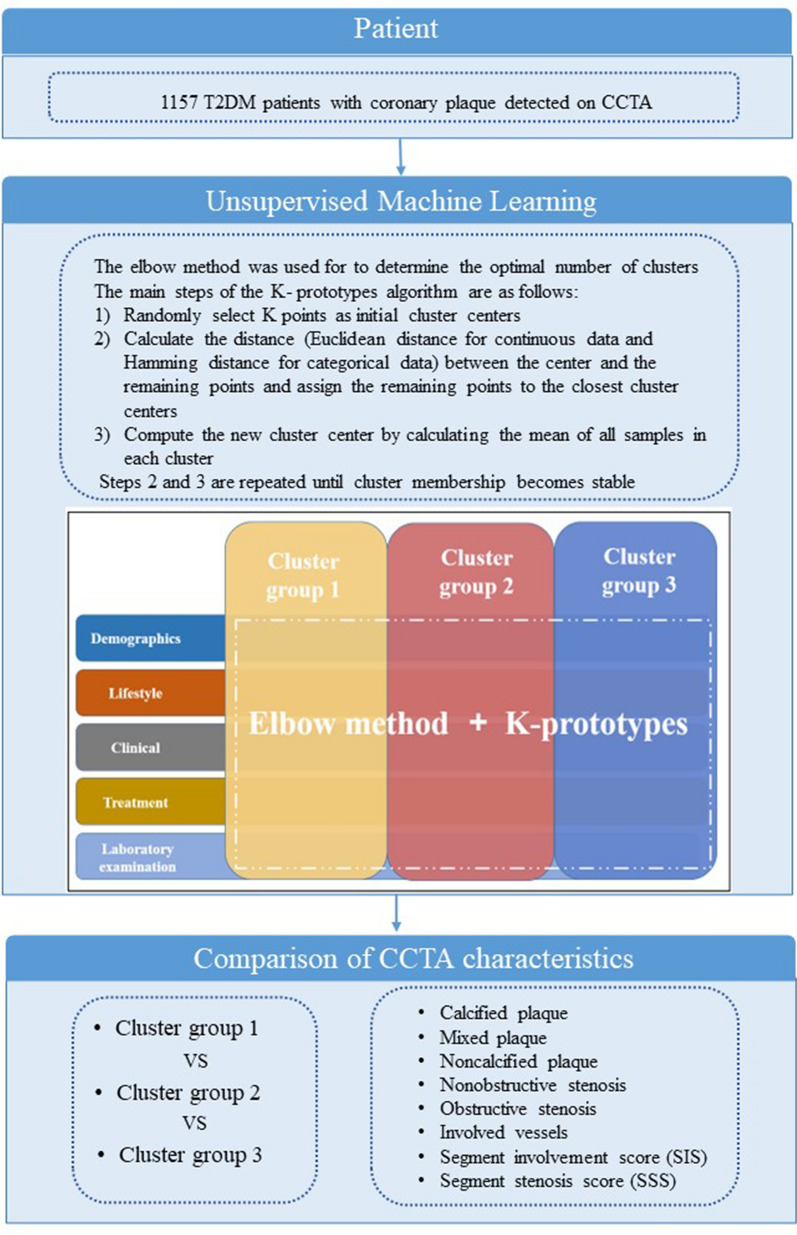
Schematic for the main steps of this study

The K-prototypes algorithm with the elbow method was used for clustering of the 1157 T2DM patients based on clinical characteristics (Fig. 2). The elbow method was used for to determine the optimal number of clusters. The core idea of the elbow method is to minimize the sum of the squared error between the cluster center and the remaining points of the corresponding clusters. As the cluster number (K) increases, the separation of the model is more distinguished. When K increases beyond the optimal value, the sum of the squared error will not substantially change. K-prototype clustering is a method that combines K-means and K-modes for clustering objects mixed with continuous and categorical data [21]. The main steps of the K-prototypes algorithm are as follows: (1) Randomly select K points as initial cluster centers; (2) Calculate the distance (Euclidean distance for continuous data and Hamming distance for categorical data) between the center and the remaining points and assign the remaining points to the closest cluster centers; and (3) Compute the new cluster center by calculating the mean of all samples in each cluster. Steps 2 and 3 are repeated until cluster membership becomes stable. The unsupervised clustering method allowed the optimal number of clusters to explain the overall variance of the data to be determined (cluster group 1: n = 463, cluster group 2: n = 341, cluster group 3: n = 353). The differences in clinical variables and the characteristics of coronary atherosclerosis among the three cluster subgroups were analyzed and compared. The scikit-learn library (version 0.24.2) based on Python (version 3.7.0) was used for the K-prototypes clustering with elbow method.

### Statistical analysis

After the identification of the three cluster groups, clinical information and coronary atherosclerosis characteristics were compared among the cluster groups. All statistical analyses were performed using SPSS software (version 25.0; IBM, Armonk, New York, USA). Categorical variables are presented as numbers (%), and continuous variables are expressed as the mean ± standard deviation in this study. The comparison of clinical information and coronary atherosclerosis characteristics among cluster groups in categorical variables were compared using Fisher’s exact tests (when expected cell value ≤ 5) or the chi-square test followed by Bonferroni’s post hoc test. The Kruskal–Wallis rank test or one-way analysis of variance followed by Bonferroni’s post hoc test was used to compare continuous data among cluster groups. A two-tailed P value of less than 0.05 was considered indicative of statistical significance.

## Result

### Study population

A total of 1157 T2DM individuals were included in the study, of whom 65.7% (760/1157) were men, with an average age of 69.32 ± 9.89 years old. Based on the 27 clinical parameters of the 1157 T2DM participants, the unsupervised analysis identified three subgroups of T2DM patients with coronary atherosclerosis, which were named cluster group 1 (n = 463), cluster group 2 (n = 341) and cluster group 3 (n = 353). The main clinical characteristics of the participants in the three cluster groups are shown in Table 1.

**Table 1:** Baseline characteristics of the study cohort

	Cluster 1 (n = 463)	Cluster 2 (n = 341)	Cluster 3 (n = 353)	P value
Male (%)	193(41.7%)	226(66.3%)^a^	341(96.6%)^a,b^	< 0.001
Age (years old)	71.44 ± 9.21	69.98 ± 9.56	65.89 ± 10.15^a,b^	< 0.001
BMI (kg/m^2^)	24.69 ± 3.51	24.71 ± 3.38	24.64 ± 3.26	0.856
Smoking history (%)	0(0.0%)	130(38.1%)^a^	322(91.2%)^a,b^	< 0.001
Alcohol (%)	4(0.9%)	96(28.2%)^a^	211 (59.8%)^a,b^	< 0.001
Hypertension (%)	389(84.0%)	254(74.5%)^a^	280(79.3%)	0.004
Systolic blood pressure (mmHg)	137.71 ± 19.45	136.04 ± 20.27	136.48 ± 20.69	0.297
Diastolic blood pressure (mmHg)	79.82 ± 12.61	77.60 ± 11.51	81.31 ± 13.30^b^	0.001
Pulse pressure (mmHg)	57.89 ± 17.13	58.43 ± 17.66	55.16 ± 15.33^b^	0.028
CAD family history (%)	19(4.1%)	14(4.1%)	30(8.5%)^a^	0.010
Diabetes duration (year)	9.94 ± 7.29	7.90 ± 8.20^a^	9.70 ± 7.37^b^	< 0.001
HbA1c (%)	7.41 ± 1.48	7.52 ± 1.65	7.62 ± 1.54	0.158
Fasting blood glucose (mmol/L)	7.50 ± 2.60	7.99 ± 2.91	7.56 ± 2.58	0.064
Cholesterol(mmol/L)	3.96 ± 1.18	4.06 ± 1.12	3.89 ± 1.06	0.175
Triglyceride(mmol/L)	1.58 ± 1.08	1.64 ± 1.28	1.65 ± 1.02	0.124
HDL-C(mmol/L)	1.16 ± 0.31	1.12 ± 0.35	1.07 ± 0.28^a,b^	< 0.001
LDL-C(mmol/L)	2.22 ± 0.93	2.32 ± 0.92	2.23 ± 0.87	0.298
Serum uric acid (µmol/L)	318.97 ± 97.29	328.26 ± 98.99	337.27 ± 90.12^a^	0.014
eGFR(ml/min/1.73m^2^)	78.75 ± 17.08	78.72 ± 16.95	83.81 ± 19.06^a,b^	< 0.001
Serum creatinine (µmol/L)	74.46 ± 22.18	79.79 ± 21.97^a^	81.77 ± 23.20^a^	< 0.001
Diabetes treatment (%)				
Oral	463(100.0%)	0(0.0%)^a^	353(100.0%)^b^	< 0.001
Biguanides	257(55.5%)	0(0.0%)^a^	217(61.5%)^b^	< 0.001
α-Glucosidase inhibitor	153(33.0%)	0(0.0%)^a^	137(38.8%)^b^	< 0.001
Sulfonylureas	123(26.6%)	0(0.0%)^a^	84(23.8%)^b^	< 0.001
Insulin	119(25.7%)	122(35.8%)^a^	101(28.6%)	0.007
Without drug	0(0.0%)	197(57.8%)^a^	0(0.0%)^b^	< 0.001
Statins (%)	123(26.6%)	60(17.6%)^a^	97(27.5%)^b^	0.003

^a^Indicates that the difference between cluster group 2 or cluster group 3 and cluster group 1 is statistically significant

^b^Indicates that the difference between cluster group 3 and cluster group 2 is statistically significan t

### Cluster group 1

The results showed that cluster group 1 had the fewest male patients (41.7%), the lowest proportion of T2DM patients with smoking (0%) or alcohol drinking history (0.9%), and the lowest level of serum creatinine (74.46 ± 22.18 µmol/L). Compared with cluster 2, patients in cluster 1 were more likely to have hypertension (84%), had a longer duration of diabetes (9.94 ± 7.29 years), and had a higher proportion of patients who underwent diabetes treatment. Compared with cluster 3, patients in cluster 1 had a lower proportion of patients with CAD family history (4.1%) and had lower levels of serum uric acid (318.97 ± 97.29 µmol/L).

### Cluster group 2

The patients in cluster group 2 had the shortest duration of diabetes (7.90 ± 8.20 years) and had the lowest proportion of patients who underwent diabetes treatment (42.2%) or were treated with statins (17.6%). The patients in cluster group 2 had a lower proportion of hypertension (74.5%) than those in cluster 1, and had a lower level of diastolic blood pressure (77.60 ± 11.51 mmHg) and a higher level of pulse pressure (58.43 ± 17.66 mmHg) those in cluster 3.

### Cluster group 3

The patients in cluster group 3 were the youngest (65.89 ± 10.15 years old), had the highest proportion of male patients (96.6%), had the highest proportion of patients with a history of smoking (91.2%) and alcohol drinking (59.8%), had the highest level of eGFR (83.81 ± 19.06 ml/min/1.73m^2^), and had the lowest level of HDL-C (1.07 ± 0.28 mmol/L).

### Association of cluster identity with coronary artery atherosclerosis

**Fig. 3:**
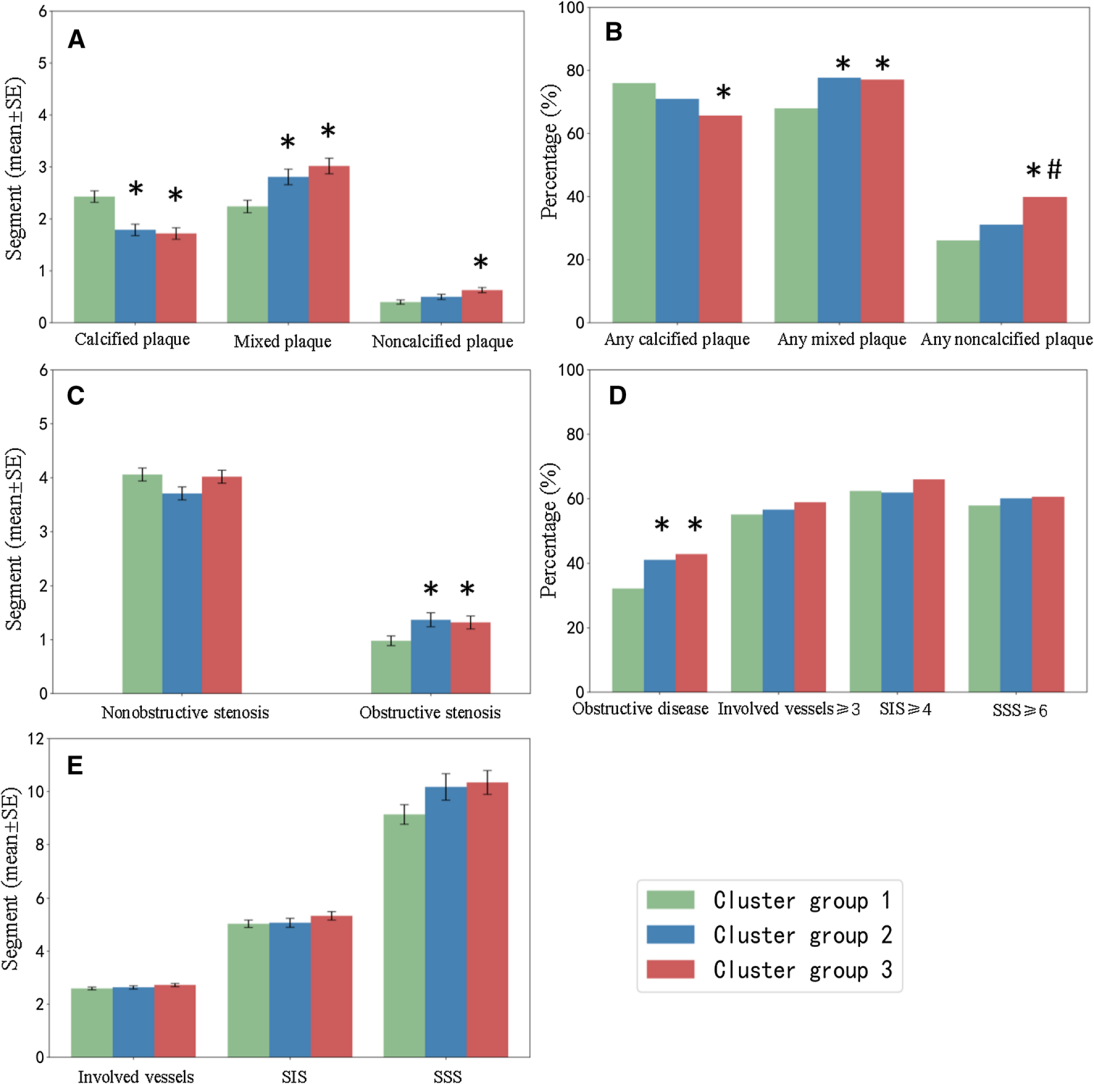
Characteristics of coronary artery atherosclerosis among the three clustering groups. **A** The number of coronary artery segments (mean value ± standard error [SE]) of different types of plaque; **B** percentage of T2DM patients with each type of plaque; **C** the number of coronary artery segments with obstructive and nonobstructive coronary artery stenosis (mean value ± SE); **D** the percentage of T2DM patients with obstructive coronary disease, ≥ 3 involved vessels, segment involvement score (SIS) ≥ 4, or segment stenosis score (SSS) ≥ 6; **E** the number of diseased vessels, SIS and SSS (mean value ± SE). ^*^Denotes that the difference between cluster group 2 or cluster group 3 and cluster group 1 is statistically significant. ^#^Denotes that the difference between cluster group 3 and cluster group 2 is statistically significant

The characteristics of coronary artery atherosclerosis among the three clustering groups are compared in Table 2 and Fig. 3.

**Table 2:** Comparison of coronary artery plaque characteristics of three cluster groups in T2DM

	Cluster 1 (n = 463)	Cluster 2 (n = 341)	Cluster 3 (n = 353)	P value
Segments of different plaque types				
Calcified plaque	2.43 ± 2.46	1.79 ± 1.96^a^	1.72 ± 1.98^a^	< 0.001
Mixed plaque	2.24 ± 2.59	2.81 ± 2.78^a^	3.02 ± 2.84^a^	< 0.001
Noncalcified plaque	0.40 ± 0.78	0.50 ± 0.95	0.63 ± 1.02^a^	< 0.001
Segments with different degrees of stenosis				
Minimal stenosis	2.13 ± 1.78	1.81 ± 1.58	2.00 ± 1.70	0.069
Mild stenosis	1.91 ± 2.05	1.89 ± 1.84	1.99 ± 2.01	0.619
Moderate stenosis	0.72 ± 1.48	0.89 ± 1.58	0.91 ± 1.57^a^	0.020
Severe stenosis	0.26 ± 0.91	0.48 ± 1.43	0.40 ± 1.09^a^	0.021
Non obstructive stenosis	4.06 ± 2.47	3.71 ± 2.21	4.02 ± 2.34	0.189
Obstructive stenosis	0.98 ± 2.00	1.37 ± 2.45^a^	1.32 ± 2.17^a^	0.003
Involved vessels	2.60 ± 1.07	2.64 ± 1.09	2.73 ± 1.03	0.275
SIS	5.04 ± 3.05	5.08 ± 3.10	5.34 ± 3.05	0.278
SSS	9.14 ± 7.96	10.18 ± 9.24	10.35 ± 8.51	0.121
Patient level				
Patients with calcified plaques	352(76.0%)	242(71.0%)	232(65.7%)^a^	0.005
Patients with mixed plaque	315(68.0%)	265(77.7%)^a^	272(77.1%)^a^	0.002
Patients with noncalcified plaque	121(26.1%)	106(31.1%)	141(39.9%)^a,b^	< 0.001
Obstructive disease	149(32.2%)	140(41.1%)^a^	151(42.8%)^a^	0.003
Involved vessels ≥ 3	255(55.1%)	193(56.6%)	208(58.9%)	0.546
SIS ≥ 4	289(62.4%)	211(61.9%)	233(66.0%)	0.458
SSS ≥ 6	268(57.9%)	205(60.1%)	214(60.6%)	0.693

^a^Indicates that the difference between the two groups is statistically significant

^b^Indicates that the difference between the two groups is statistically significant

In terms of plaque types (Fig. 3A), cluster group 1 had the largest number of segments with calcified plaques (2.43 ± 2.46) and the smallest number of segments with mixed plaques (2.24 ± 2.59). Cluster group 3 had more segments with noncalcified plaques than cluster group 1 (cluster group 3 vs. cluster group 1: 0.63 ± 1.02 vs. 0.40 ± 0.78, P < 0.05). At the patient level (Fig. 3B), cluster group 3 had the highest proportion of patients with noncalcified plaques (39.9%). Cluster group 1 had the lowest proportion of patients with mixed plaques (68.0%). Cluster group 3 had a lower proportion of patients with calcified plaques than cluster 1 (65.7% vs. 76.0%, P < 0.05).

In terms of the degree of coronary artery stenosis (Fig. 3C), cluster group 1 had the least number of segments with obstructive stenosis (0.98 ± 2.00). Cluster group 3 had more segments with moderate stenosis (0.91 ± 1.57 vs. 0.72 ± 1.48) and severe stenosis (0.40 ± 1.09 vs. 0.26 ± 0.91) than cluster group 1 (P values < 0.05). At the patient level, cluster group 1 had the lowest proportion of patients with obstructive stenosis (32.2%) (Fig. 3D).

There was no significant difference in involved vessels, SIS, SSS or proportions of involved vessels ≥ 3, SIS ≥ 4, and SSS ≥ 6 among the three cluster groups (all P values > 0.05) (Fig. 3E).

## Discussion

In this study, an unsupervised machine learning method was used to explore the subgroups of T2DM patients with different clinical characteristics. The unsupervised machine learning method provides techniques to integrate various data to enable the discovery of new biomarkers without providing specifications about how to partition the data based on expertise [17, 22]. Our data demonstrated that unsupervised machine learning methods could be used to address heterogeneous clinical data and have the potential to distinguish among subgroups of T2DM patients with different plaque types and degrees of coronary artery stenosis.

### Unsupervised machine learning for processing clinical data

It is known that aging, diabetes, hypertension, hyperlipidemia, or declining renal function alone is a risk factor for coronary artery disease [4, 23, 24]. Currently, the combined effects of these factors on coronary artery disease are gaining more attention. Previous research focused mainly on the combination of two or three factors for coronary artery disease [25–27]. Unsupervised machine learning for clustering does not rely on manual judgement; thus, this method may have the potential to distinguish a group of patients with similar clinical situations. As the clinical data grow rapidly, the clustering method may be more useful for processing the various and heterogeneous data in electronic clinical records.

### Relationship of coronary plaque types with clusters

We observed that the clustering method could not only distinguish T2DM patients with different clinical contexts, but also indirectly identify the group with different types of coronary plaque. The results showed that cluster 3 had relatively more segments with mixed and noncalcified plaques. This may be explained by the fact that cluster group 3 had the highest proportion of males, tended to have the unhealthy habits, including smoking and alcohol drinking, and had the lowest level of HDL-C. Current smoking has been reported to be a risk factor for mixed and noncalcified plaques in coronary atherosclerosis [28]. Mild to moderate alcohol consumption may reduce cardiovascular risk, while heavy drinking may promote to coronary artery calcification [29]. Treatment to raise HDL-C levels regressed coronary plaque and reduced lipid content in plaque [30]. A previous study indicated that the presence of mixed plaques and noncalcified plaques had relatively higher risks of cardiovascular events than calcified plaques [31]. This result draws attention to the importance of proper management for T2DM patients with these risk factors for coronary plaques.

### Obstructive coronary disease in clusters

Cluster 1 had the least number of obstructive coronary stenosis cases and the lowest proportion of patients with obstructive coronary disease in this study. This may in part be explained by the fact that cluster 1 had the lowest proportion of males and was less likely to smoke or drink alcohol. A previous study showed that males had a larger plaque volume than females [32]. A multicenter prospective CCTA cohort study showed a similar result: obstructive coronary disease was more prevalent in men than in women (42% vs. 26%) [33]. It has been reported that coronary artery stenosis in T2DM patients adds to the risk of an acute plaque event [34]. Another study also demonstrated that obstructive coronary artery disease was a predictor of cardiac events in diabetic patients [35]. We also noticed that patients in cluster 3 were the youngest. Traditionally, aging is considered to be a risk factor for coronary atherosclerosis. From this, we deduce that cluster 3 would have more obstructive disease when adjusted for age. It also emphasizes the necessity of early diagnosis and timely treatment for T2DM patients with obstructive coronary disease to reduce cardiac events.

### Association of extent of coronary atherosclerosis with clusters

Although there were some differences in coronary plaque types and the degree of luminal stenosis, there was no significant difference in the extent of coronary atherosclerosis among the three cluster groups. a previous study demonstrated that the risk of mortality usually correlates with the extent of coronary atherosclerosis in individuals with and without T2DM [36]. Another study showed that atherosclerotic risk factors such as systolic blood pressure, LDL-C, and current smoking have heterogeneous impacts on arterial territories of different vascular diseases, including coronary ischemic and hemorrhagic stroke, abdominal aortic aneurysms, and peripheral arterial disease [37]. We may infer that the extent of coronary atherosclerosis was affected by various factors. Whether and how the different combinations of clinical data with similar coronary characterization affect prognosis are not entirely clear. Thus, the comprehensive effect of the risk factors on coronary atherosclerosis will be investigated in more detail in future studies to verify the benefits of the comprehensive assessment.

## Limitations

There are some limitations of this study. First, as a single-center study, selection bias is inevitable, and multicenter studies are needed to verify the results in the future. Second, this was a retrospective study, and follow-up information was not included. The evolution of coronary atherosclerosis in T2DM patients requires further exploration. Third, coronary artery stenosis evaluated with CCTA in this study was not validated with coronary angiography. However, CCTA, a noninvasive examination, can be used to evaluate different plaque types as well as luminal stenosis and has been widely accepted in the evaluation of coronary plaque [38].

## Conclusions

The unsupervised clustering method could address T2DM patients with heterogeneous clinical indicators, which may contribute to the stratification of T2DM patients with coronary atherosclerosis. Our findings show one potential way to identify T2DM patients with different types of coronary plaque and degrees of coronary stenosis based on clinical data, which is essential for the clinical management of T2DM patients with coronary atherosclerosis.

## Data Availability

The datasets used and analyzed during the current study are available from the corresponding author on reasonable request.

## Abbreviations

T2DM: Type 2 diabetes mellitus
CCTA: Coronary computed tomography angiography
SIS: Segment involvement score
SSS: Segment stenosis score
eGFR: Estimated glomerular filtration rate
HDL-C: High-density lipoprotein cholesterol
